# Pre-operative Mental Health and Adverse Outcomes Following Total Knee Replacement: A Prospective Cohort Study

**DOI:** 10.1192/bjo.2024.84

**Published:** 2024-08-01

**Authors:** Jonathan Gibb, Erik Lenguerrand, Vikki Wylde, Krishnan Bhaskaran, Michael Whitehouse

**Affiliations:** 1 Centre for Academic Mental Health, Department of Population Health Sciences, Bristol Medical School, University of Bristol, Bristol, United Kingdom; 2 The London School of Hygiene & Tropical Medicine, London, United Kingdom; 3 Musculoskeletal Research Unit, University of Bristol, Bristol, United Kingdom

## Abstract

**Aims:**

Total knee replacements (TKRs) are effective procedures for severe osteoarthritis. Some studies suggest that people with common pre-operative mental health problems are more likely to experience complications following joint replacement. This study aimed to determine whether people who described pre-operative anxiety or depression were more likely to report an adverse event, or outcome, following a TKR.

**Methods:**

A prospective cohort of people undergoing TKR at a surgical centre in England between 2012–2013 as part of service evaluation were studied. Following informed consent, participants completed pre-operative sociodemographic questionnaires alongside several patient-reported outcome measures (PROMs): the Western Ontario and McMaster Universities Osteoarthritis Index (WOMAC), EuroQoL Five-dimensions Descriptive System (EQ-5D-3L), and the Self-Administered Patient Satisfaction Scale for Primary Knee Arthroplasty. Participants were classified as exposed if they described moderate or extreme problems with anxiety or depression in the mental health subset of the EQ-5D-3L. The primary outcome was the presence of a patient-reported adverse event (bleeding, infection or fracture) at 3 months post-surgery measured through a short postal questionnaire. Repeat PROMs were assessed at 3- and 12-months post-surgery. Logistic regression was used to model the association between pre-operative mental health status and probability of an adverse event, or outcome, occurring following adjustment for age, sex and body mass index.

**Results:**

Of the 206 individuals studied, over a third (n 72/206, 35%) had reported problems with anxiety or depression before surgery. Among those returning completed follow-up questionnaires, 20% (n 34/168) described an adverse event at 3 months. Pre-operative anxiety or depression was not associated with an increased odds of reporting an adverse event (aOR 0.85, 95% CI 0.35–2.05) at 3 months post-surgery. People who described problems with anxiety or depression were more likely to have a greater degree of pre-operative functional impairment. Even after adjusting for a higher pre-operative symptom burden, exposed participants were more likely to report problems with activities of daily living (aOR 2.32, 95% CI 1.09–4.94) and pain or discomfort (aOR 5.58, 95% CI 1.77–17.60) at 3 months post-surgery. However, they did not have an increased odds of describing worse function, reduced health-related quality of life, or being dissatisfied with their TKR at 12 months post-surgery.

**Conclusion:**

Despite having a higher burden of morbidity prior to undergoing surgery, pre-operatively anxious or depressed participants did not have an increased odds of reporting an adverse event at 3 months and went on to experience comparable improvements in PROMs at 12 months post-surgery.

